# Betulinic acid shows anticancer activity against equine melanoma cells and permeates isolated equine skin in vitro

**DOI:** 10.1186/s12917-020-2262-5

**Published:** 2020-02-05

**Authors:** Lisa A. Weber, Jessica Meißner, Julien Delarocque, Jutta Kalbitz, Karsten Feige, Manfred Kietzmann, Anne Michaelis, Reinhard Paschke, Julia Michael, Barbara Pratscher, Jessika-M. V. Cavalleri

**Affiliations:** 1 Clinic for Horses, University of Veterinary Medicine Hannover, Foundation, Bünteweg 9, 30559 Hannover, Germany; 2 Department of Pharmacology, Toxicology and Pharmacy, University of Veterinary Medicine Hannover, Foundation, Bünteweg 17, 30559 Hanover, Germany; 3 Biosolutions Halle GmbH, Weinbergweg 22, 06120 Halle (Saale), Germany; 4 Biozentrum, Martin Luther University Halle-Wittenberg, Weinbergweg 22, 06120 Halle (Saale), Germany; 5 Skinomics GmbH, Weinbergweg 23, 06120 Halle (Saale), Germany; 6 University Small Animal Clinic, University of Veterinary Medicine Vienna, Veterinärplatz 1, 1210 Vienna, Austria; 7 University Equine Clinic, University of Veterinary Medicine Vienna, Veterinärplatz 1, 1210 Vienna, Austria

**Keywords:** Equine malignant melanoma (EMM), Betulinic acid, Cell culture assay, Franz-type diffusion cell

## Abstract

**Background:**

Equine malignant melanoma (EMM) is a frequently occurring dermoepidermal tumor in grey horses. Currently available therapies are either challenging or inefficient. Betulinic acid (BA), a naturally occurring triterpenoid, is a promising compound for cancer treatment. To evaluate the potential of BA as a topical therapy for EMM, its anticancer effects on primary equine melanoma cells and dermal fibroblasts and its percutaneous permeation through isolated equine skin were assessed in vitro.

**Results:**

BA showed antiproliferative and cytotoxic effects on both primary equine melanoma cells and fibroblasts in a time- and dose-dependent manner. The lowest half-maximal inhibitory concentrations were obtained 96 h after the beginning of drug exposure (12.7 μmol/L and 23.6 μmol/L for melanoma cells eRGO1 and MelDuWi, respectively, in cytotoxicity assay). High concentrations of the compound were reached in the required skin layers in vitro.

**Conclusion:**

BA is a promising substance for topical EMM treatment. Further clinical studies in horses are necessary to assess safety and antitumoral effects in vivo*.*

## Background

Betulinic acid (BA), a naturally occurring pentacyclic triterpenoid in the bark of plane and birch trees, has been demonstrated to exert a variety of biological features. In addition to its anti-HIV [1], antiparasitic [2] and anti-inflammatory [3] properties, BA shows anticancer activity in vitro and in vivo [4–10]. Its antitumor effects are mediated mainly by a CD95- and p53-independent induction of apoptosis [11]. Formation of the mitochondrial permeability transition pore complex leads to cytochrome *c* and apoptosis-inducing factor release with subsequent caspases activation [12, 13]. Further molecular antitumoral mechanisms, such as reactive oxygen species formation [14, 15], mitogen-activated protein kinase activation [16], angiogenesis inhibition [17, 18] and other controlled cell death mechanisms [19], have been implicated. Moreover, a selective cytotoxicity on human cancer cells compared to normal cells has been described [5, 20, 21] and might be explained by BA’s ability to inhibit the steroyl-CoA-desaturase activity [22]. As tumor cells depend on de novo lipogenesis but not normal cells, inhibition of this enzyme leads to enhanced saturation levels of mitochondrial cardiolipins. Hence, ultrastructural changes in the mitochondrial membrane and subsequent release of cytochrome *c* cause cell death [22]. BA’s ability to induce apoptosis has also been demonstrated in equine melanoma cells in vitro [23].

Equine malignant melanoma (EMM) is a common skin neoplasm in aging grey horses [24–26]. An intronic mutation in the STX17 (syntaxin-17) gene was identified as a link to the grey horse phenotype and predisposition to melanoma [27, 28]. EMMs are firm, mostly spherical, occasionally ulcerated tumors of various size arising from the melanocytes mainly in glabrous cutaneous regions [25]. Predilection sites are the ventral surface of the tail, perineal region, external genitalia, eyelids and lips [29, 30]. Additionally, they are commonly found in the guttural pouch and parotid gland [31]. It has been reported that melanomas represent 3.8% of neoplastic diseases in horses [32]. EMMs progress to malignancy in more than 60% of cases and can cause widespread visceral metastases [31, 33–35]. While some lesions do not cause any clinical problems, others can lead to impaired defecation, colic, weight loss, edema, keratitis and ataxia, depending on the location and size of the tumor [31, 36, 37]. Currently available therapies are either inefficient or challenging. Immunological therapeutic approaches are promising [38] but require further research. Hence, local treatment modalities such as surgical excision, and chemotherapeutic drugs like intralesional cisplatin are commonly used [39–42]. However, unfavorable tumor location might prohibit surgical excision in many cases and the cytotoxic agent cisplatin entails toxic drug exposure risk for the treating veterinarian and any other person coming in contact with the substance (e.g. horse owner, groom) [42]. Thus, more feasible topical treatment options for EMM should be considered. Therefore, the objectives of this study are (1) to assess the antiproliferative and cell viability reducing effects of BA on primary equine melanoma cells and primary equine fibroblasts, (2) to demonstrate a selective cytotoxicity to equine melanoma cells, and (3) to investigate the penetration and permeation ability of BA in a pharmaceutical test formulation on isolated equine skin in vitro.

## Results

### Cell characterization

**Fig. 1:**
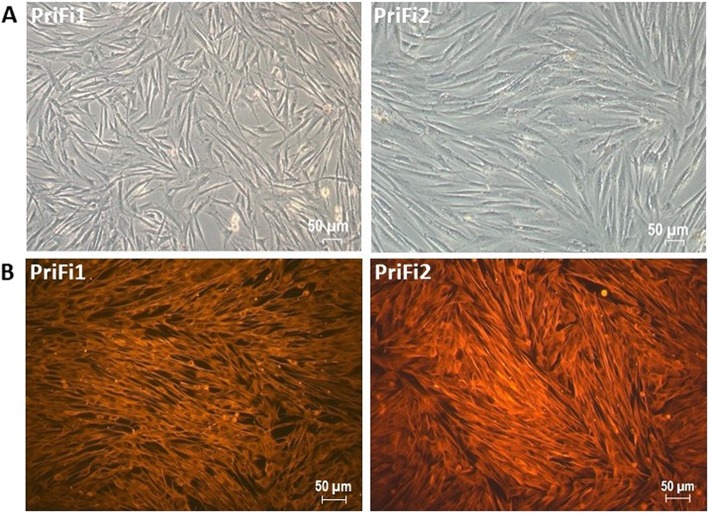
Verification of dermal fibroblasts (PriFi1 and PriFi2) isolated from the skin of two different horses. **a** Phase contrast microscopy of primary equine dermal fibroblasts PriFi1 and PriFi2. Cells show a typical spindle-shaped morphology. × 10 magnification. **b** Positive fluorescence microscopy detection of intermediate filament vimentin (red fluorescence) in PriFi1 and PriFi2. × 20 magnification, 546 nm

Indirect immunocytochemistry was performed to characterize the primary equine dermal fibroblasts. PriFi1 and PriFi2 stained positive for vimentin (Fig. 1), whereas no signal was detected after incubation with anti-cytokeratin. These results, in combination with the spindle-shaped cell morphology, verified PriFi1 and PriFi2 as fibroblasts.

### Proliferation inhibition and cytotoxicity of BA on equine cells

**Fig. 2:**
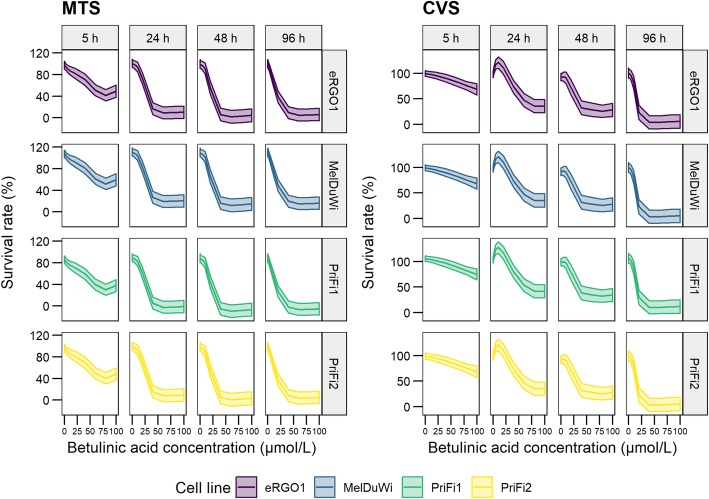
Predicted mean values and 95% confidence intervals of the survival rates for different equine cells. eRGO1 and MelDuWi = primary equine melanoma cells, PriFi1 and PriFi2 = primary equine dermal fibroblasts**.** Cytotoxic effects investigated by MTS assay, antiproliferative effects determined by CVS assay. Data represent predicted mean values and 95% confidence intervals of 6–8 independent experiments for each combination of cell type, incubation time and concentration as given by the generalized additive models. BA had a stronger cytotoxic effect when cells were exposed for 24, 48 and 96 h compared to 5 h (*P* < 0.001 each). While there was a highly significant difference in cytotoxicity between 24 h and 96 h (P < 0.001), cytotoxic effects differed less between 24 h and 48 h (*P* < 0.01) and 48 h and 96 h (*P* < 0.05). Equally, there was a statistically significant difference in the cell proliferation between a treatment duration of 5 h compared to 24, 48 and 96 h (P < 0.001 each). A treatment duration of 24 h compared to 48 h, 24 h compared to 96 h and 48 h compared to 96 h revealed a high significance in cell proliferation (P < 0.001 each). A pairwise comparison of all cell types revealed PriFi1 as the most sensitive cell type in MTS assay (P < 0.001 for PriFi1 vs. all other cell types), whereas it was the most resistant one in CVS (P < 0.001 for PriFi1 vs. all other cell types). MelDuWi was the most resistant cell type towards BA’s cytotoxic effects (P < 0.001 for MelDuWi vs. all other cell types). In conclusion, betulinic acid did not show a selectivity to equine melanoma cells compared to normal cells

The antiproliferative and cytotoxic effects of BA on primary equine melanoma cells and primary equine dermal fibroblasts were investigated. The compound had significant effects on the inhibition of cell proliferation (*P* < 0.001 for CVS for every duration of incubation) and the reduction of cell viability (P < 0.001 for MTS for every duration of incubation) on both equine melanoma cells and fibroblasts in a dose-dependent manner. With increasing treatment duration, cell proliferation and cell viability decreased significantly (Fig. 2). A selectivity of the compound to tumor cells compared to normal cells could not be demonstrated (Fig. 2). When cells were exposed to the drug for 5 h, the quantity of cells affected was too low to calculate the IC_50_ values in both cytotoxicity and proliferation assays. The lowest IC_50_ values for all cells were obtained in both, cytotoxicity and proliferation assays, 96 h after the beginning of drug exposure (Table 1).

**Table 1:** IC_50_ values (μmol/L) of betulinic acid for primary equine cells determined by CVS and MTS assay

cells	24 h	48 h	96 h
MTS assay			
eRGO1	22.8 (−3–48)	20.7 (13–29)	12.7 (11–15)
MelDuWi	34.6 (24–45)	31.7 (25–38)	23.6 (13–34)
PriFi1	20.4 (19–22)	18.0 (17–19)	13.8 (7–21)
PriFi2	24.8 (11–39)	22.7 (1–49)	13.3 (11–16)
Crystal violet staining assay			
eRGO1	25.9 (20–32)	21.2 (− 2–44)	19.6 (11–29)
MelDuWi	49.2 (31–67)	35.8 (− 22–94)	21.6 (5–38)
PriFi1	58.0 (52–64)	52.2 (39–65)	14.5 (14–15)
PriFi2	30.3 (17–44)	29.1 (6–53)	13.8 (10–18)

Cytotoxic (MTS assay) and antiproliferative (crystal violet staining assay) effects of betulinic acid on primary equine melanoma cells (eRGO1 and MelDuWi) and primary equine dermal fibroblasts (PriFi1 and PriFi2) after a treatment duration of 24, 48, or 96 h. Data represent mean IC_50_ values (μmol/L) of 6–8 independent experiments with 95% confidence interval in parentheses

### Diffusion of BA into equine skin and overall BA recoveries

**Fig. 3:**
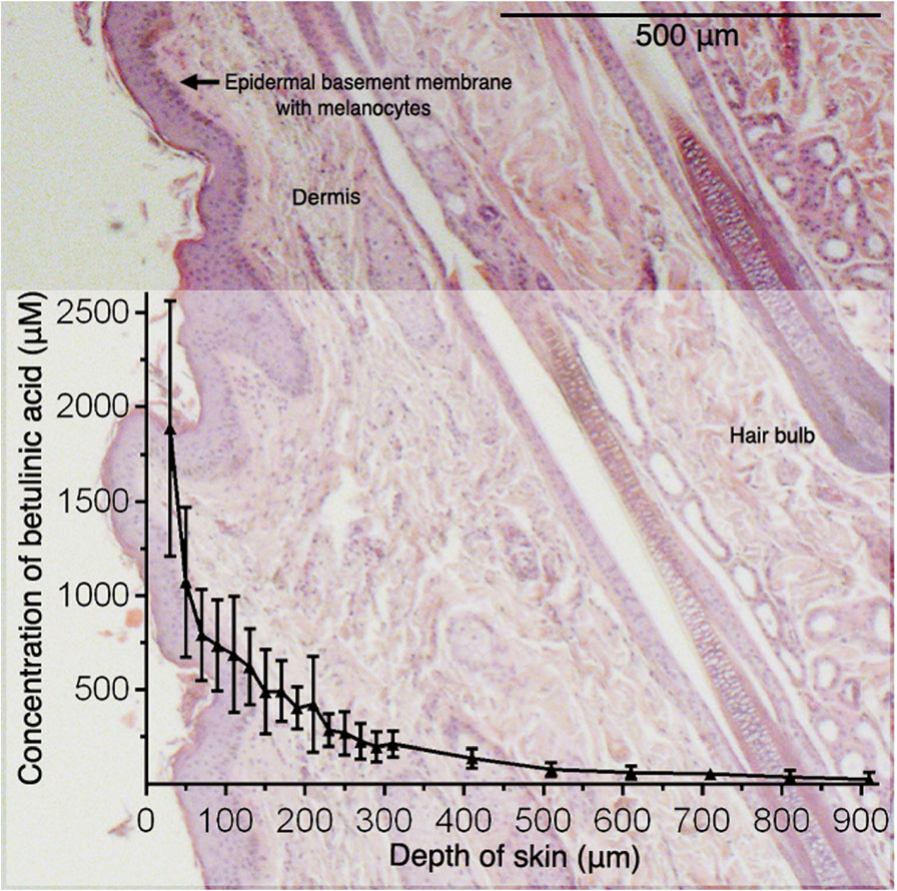
Concentration profile of betulinic acid correlative to skin thickness. Thoracic skin of seven horses (two technical replicates each) were used for 24-h Franz-type diffusion cell experiments with “Basiscreme DAC” containing 1% betulinic acid and 20% medium-chain triglycerides. Data represent mean concentration (±SD) of betulinic acid in cryostat skin slices at different skin depths. Detected amounts of BA by far exceeded the determined IC_50_ values for equine melanoma cells after 24 h, especially in the uppermost skin layers (410 μm). As minor cream residues on the skin surface after cleaning with a cotton swab cannot be excluded, data for 10 μm skin depth were eliminated in this figure. Hematoxylin and eosin staining of equine lateral thoracic skin kindly provided by the Institute for Anatomy, University of Veterinary Medicine Hannover, Foundation, Hannover, Germany

The penetration and permeation properties of 1% BA with 20% medium-chain triglycerides in “Basiscreme DAC” on isolated equine skin using FDCs were evaluated to identify an effective formulation for prospective in vivo use. An overall BA recovery of 98 ± 7% (mean ± SD; *n* = 7) was achieved. A quantity of 18 ± 11% of the amount of BA applied was detected in the acceptor media and 56 ± 13% in the cotton swabs. In the skin, 24 ± 1% of the BA amount applied was analyzed, from which 9 ± 7% were found in the blade cleaning tissues. BA was able to penetrate the *stratum corneum* and permeate through the epidermal and dermal layers of the isolated equine skin within 24 h (Fig. 3). At a depth of 810 μm, the concentration of BA was still 39.6 μmol/L ± 38 μmol/L (mean ± SD). Including this skin layer, the BA concentration in isolated equine skin exceeded the 24-h IC_50_ values of both equine melanoma cells and fibroblasts investigated by the cytotoxicity assay in all layers examined. Up to a depth of 710 μm, the 24-h IC_50_ values of equine melanoma cells investigated by proliferation assay were surpassed (55.8 μmol/L ± 31 μmol/L).

## Discussion

The aim of these in vitro studies was to explore the potential of BA as a topical therapy against EMM. Antiproliferative and cytotoxic effects of the compound on primary equine melanoma cells and primary equine dermal fibroblasts as well as its permeation through isolated equine skin were investigated. It could be shown that BA inhibits proliferation and cell metabolism in equine melanoma cells and dermal fibroblasts in a time- and dose-dependent manner. Moreover, when 1% BA in “Basiscreme DAC” supplemented with 20% medium-chained triglycerides was applied on isolated equine skin, high concentrations of the compound were reached in the required skin layers.

Antiproliferative and cytotoxic effects were observable as early as after 5 h of drug exposition, however, at this time point the quantity of cells affected was too low to calculate IC_50_ values. The results demonstrate that antiproliferative and cytotoxic effects increase with treatment duration and thus the lowest IC_50_ values were obtained 96 h after the beginning of drug exposure. With the four different incubation time points and the resulting IC_50_ values, information about the time-dependent cytotoxic and antiproliferative effects of BA on equine cells were added - not only after 96 h, as reported previously for equine melanoma cells [23], but also after 5, 24 and 48 h. This information may be valuable for the design of treatment regimes in further in vivo studies. Previously reported IC_50_ values of equine melanoma cells determined by the sulforhodamine B assay (33.1 μmol/L (MelDuWi) and 33.4 μmol/L (MelJess)) [23] were higher than the ones investigated in the present study by MTS assay (23.6 μmol/L (MelDuWi) and 12.7 μmol/L (eRGO1)) after the same duration of incubation (96 h) with BA. In the MTS assay a tetrazolium salt is reduced by mitochondrial dehydrogenases to a photometrically measurable formazan product, which quantity reflects the number of living cells in culture [43]. The sulforhodamine B dye binds to protein components of fixed cells and does not distinguish between cells with an active mitochondrial metabolic rate and those without [44]. As BA mainly targets the mitochondrial pathway of apoptosis [45], the MTS assay provides an earlier detection of reduced cell viability and consequently smaller IC_50_ values compared to those formerly reported were calculated. In addition, with the crystal violet staining assay it was demonstrated that the compound is able to not only affect the cell’s metabolism, but also to inhibit the proliferation of equine melanoma cells in vitro and therefore potentially stop tumor growth in vivo.

However, the results show that normal equine dermal fibroblasts are also sensitive to BA in the concentrations investigated. These observations are in agreement with previously reported low selectivity indices of BA for normal human dermal fibroblasts [46, 47] and attenuated high glucose-induced proliferation of human cardiac fibroblasts after treatment with BA [48]. But they are in contrast to findings in other human normal cells, such as melanocytes, dermal fibroblasts and peripheral blood lymphocytes, which revealed to be more resistant to a BA treatment than cancer cells [5, 20, 21].

The in vitro cell culture studies reported here did not focus on elucidating the molecular mechanisms behind the BA-induced cell alterations. Nevertheless, it was demonstrated before that BA leads to cell cycle perturbations in equine melanoma cells with an accumulation of cells in the subG_1_-phase [23]. The same authors did demonstrate a BA-related induction of apoptosis in equine melanoma cells by AnnexinV/Propidium iodide staining and proof of caspases 3-, 8-, and 9 activation [23]. A variety of other molecular pathways are described mainly for human cancer cells [49], but need to be verified for equine cancer cells in prospective experiments. The literature about BA’s effects towards normal cells on the molecular level is limited. While inhibition of the steroyl-CoA-desaturase is a possible explanation for BA’s selectivity to some human cancer cells compared to the non-transformed human fibroblasts Co18 [22], the mechanisms behind the results shown here is not known and further studies on healthy equine cells treated with BA are needed to understand the effective mode of action.

In a clinical setting the compound needs to reach the melanoma cells in the patient to be effective. While some melanomas are ulcerated, most are covered by epidermal and dermal skin layers [50, 51]. Thus, a topically applied substance needs to penetrate the *stratum corneum*, the major barrier for transdermal drugs, and permeate through the epidermal and dermal strata. It was demonstrated that 1% BA in “Basiscreme DAC” with 20% medium-chain triglycerides fulfilled this requirement in isolated equine thoracic skin in vitro. In the FDC experiments amounts of BA were detected that by far exceeded the determined IC_50_ values for equine melanoma cells after 24 h and therefore melanomas located in the superficial and partly deeper dermal layers (up to 810 μm) could be affected by the compound. Due to practical reasons, a standardized use of nearly glabrous skin from EMM predilection sites (e.g. perineal region, external genitalia, eyelids) was not possible. This should be considered a limitation of this study. Nevertheless, others have shown that hydrocortisone, a lipophilic substance similar to BA, penetrated hairy equine thoracic skin in the same manner as nearly glabrous equine groin skin [52]. Therefore, the penetration profile of BA in equine thoracic skin compared to the skin at predilection sites can be expected to be similar.

In vitro FDC studies can be predictive for in vivo penetration and permeation data, but due to the lack of circulation they cannot provide information about the amount of a compound that is eliminated from the skin by capillary dermal blood vessels [53]. In some EMM an increased vascularization was observed [26, 51], which could lead to a higher and faster elimination of the active compound when topically applied in vivo. On the other hand, BAs’ potential to reduce angiogenesis was demonstrated in vitro and in vivo by inhibition of hypoxia-inducible factor 1α and vascular endothelial growth factor and by a negative impact on the normal growth of the capillaries in the chorioallantoic membrane assay [17, 18, 54]. Reducing the vascularization in the tumor could increase the drug concentration in this area. Further, therapeutic strategies aiming at anti-angiogenesis are reported as adjunctive therapies against melanomas in human medicine [55].

Summarizing, the potent percutaneous permeation of BA in normal skin together with its anticancer effects on equine melanoma cells suggest that this substance may exert antitumoral effects in vivo. Even if normal equine skin cells are affected by local BA treatment, inflammatory reactions are suspected to be minor, as a topical treatment of actinic keratoses with betulin, a triterpene comparable to betulinic acid, did not lead to any side effects in 14 human patients [56]. Nevertheless, to gain more insights about the therapeutic potential of BA the safety and efficacy of the compound have to be addressed on healthy and melanoma affected equine skin in vivo.

## Conclusion

The anticancer effects of BA on equine melanoma cells together with its potent transepidermal and -dermal permeation into the required skin layers make this compound a potential substance for topical melanoma treatment in horses. A selectivity to cancer cells over normal cells could not be demonstrated. In essence, this study supports the use of BA in further preclinical and clinical trials for topical EMM treatment.

## Material and methods

### Cells and culture conditions

Self-generated primary equine dermal fibroblasts PriFi1 and PriFi2 and previously isolated primary equine melanoma cells were used for the cell culture experiments. The primary equine melanoma cells MelDuWi belong to the cell culture stock of the Clinic for Horses, University of Veterinary Medicine Hannover, Foundation, Germany, while the primary equine melanoma cells eRGO1 were provided by Dr. Barbara Pratscher, Department for Small Animals and Horses, Vetmeduni Vienna, Austria. PriFi1, PriFi2 and MelDuWi were maintained as monolayers in RPMI1640 cell culture medium with stable glutamine (Biochrom GmbH, Berlin, Germany) supplemented with 15% fetal bovine serum (FBS) superior (Biochrom GmbH) and 1% penicillin and streptomycin (10,000 international units (I.U.)/mL / 10,000 μg/mL, Biochrom GmbH) at 37 °C in a humidified atmosphere with 5% CO_2_. Melanoma cells eRGO1 were cultured in Dulbecco’s modified Eagle’s high glucose w/Glutamax (4.5 g/L) cell culture medium (GIBCO-Invitrogen, Thermofisher, Darmstadt, Germany) supplemented with 10% FBS superior (Biochrom GmbH) and 1% Antibiotic-Antimycotic (100x; GIBCO-Invitrogen), containing penicillin (10,000 units/mL), streptomycin (10,000 μg/mL) and amphotericin B (25 μg/mL).

### Dermal cell isolation

Equine dermal fibroblasts were isolated as described by Mählmann [57], with some modifications. A mare (aged 10 years) and a stallion (aged 9 years) without any apparent dermatological disorders were euthanized for reasons unrelated to this study. Immediately after euthanasia, a lateral neck region caudal to the axis (C2) was prepared in accordance with standard surgical aseptic preparation methods. A piece of skin, about 2.5 × 2.5 × 1 cm, was harvested from each horse utilizing a scalpel and forceps. Subcutaneous tissue was removed and the skin was transferred into a sterile 50-mL centrifuge tube containing 15 mL fibroblast culture medium (RPMI1640 with stable glutamine (Biochrom GmbH), 20 mM HEPES (Sigma-Aldrich, Steinheim, Germany), 20% FBS superior (Biochrom GmbH), 2% penicillin and streptomycin (10,000 I.U./mL / 10,000 μg/mL, Biochrom), and 1% amphotericin B (250 μg/mL, Biochrom GmbH). After transportation at room temperature to the laboratory, the skin was washed three times in sterile phosphate-buffered saline (PBS, pH 7.4; 1 L contains 0.2 g KCl, 8.0 g NaCl, 0.2 g KH_2_PO_4_, 1.44 g Na_2_HPO_4_ × 2H_2_O and deionized water). Subsequently, the skin was refrigerated overnight at 4 °C in a sterile centrifuge tube containing 5 mg/mL dispase I (Gibco Invitrogen) diluted in 15 mL fibroblast culture medium without FBS. After 15 h, an incubation step at 37 °C with 5% CO_2_ for 2 h followed. Afterwards, the epidermis was separated from the dermis forceps. Dermal tissue was incubated for 8 h with 1 mg/mL (0.15 U/mL) collagenase A (Roche diagnostics GmbH, Mannheim, Germany) and 2 mg/mL (1.6 U/mL) dispase I (GIBCO-Invitrogen) in 15 ml fibroblast culture medium without FBS at 37 °C with 5% CO_2_. Meanwhile, the tube was agitated every 2 h. Subsequently, the sample was centrifuged at 450×g for 10 min. After the supernatant had been discarded, the cell pellet was resuspended in 5 mL fibroblast culture medium and sifted through a 70 μm filter. The cells were finally cultivated as monolayers in 25-cm^2^ tissue culture flasks at 37 °C with 5% CO_2_. After the first passage, the cells were cultivated in modified culture medium (RPMI1640 with 15% FBS and 1% penicillin and streptomycin).

### Verification of equine dermal fibroblasts

Equine dermal fibroblasts (PriFi1 and PriFi2) were verified by indirect immunofluorescence staining applying a modified reported protocol [58], except for the secondary antibody and antibody-dilutions. Briefly, a monoclonal mouse anti-vimentin antibody (Clone V-9, Sigma-Aldrich, dilution 1:200) was used. Samples incubated with a monoclonal mouse anti-cytokeratin antibody (C-11, Invitrogen, Rockford, US, dilution 1:100) and those incubated without primary antibody served as negative controls. F(ab’)2 goat anti-mouse IgG-FITC antibody (Bio-Rad Laboratories GmbH, Munich, Germany, dilution 1:200) was used for the visualization of the signals. Cells were evaluated and photographed at 546 nm and a 20 fold magnification with a Leica fluorescence microscope (Leica Microsystems, Wetzlar, Germany) and an AxioCam MRc camera (Zeiss Microscopy GmbH, Jena, Germany).

### Evaluation of proliferation and cell toxicity of betulinic acid on equine melanoma cells and equine fibroblasts

#### Pharmacological compounds

Betulinic acid (BA) was provided by Biosolutions Halle GmbH (Halle/Saale, Germany). Dimethyl sulfoxide (DMSO) (WAK-Chemie Medical GmbH, Steinbach, Germany) was used to achieve a 20 mM stock solution.

#### Proliferation assays

The inhibitory effect of BA on cell proliferation was evaluated using a modified crystal violet staining (CVS) assay [59]. In brief, cells were seeded into 96-well microtiter plates with a density of 5000 cells/well to avert confluence of the cells during the experimental period. Twenty-four hours later, the cells were treated with serial dilutions of BA dissolved in DMSO and medium at nine different concentrations ranging from 1 to 100 μmol/L. The highest concentration of DMSO solvent was 0.5% in 100 μmol/L, which had neither an impact on the cell proliferation rate nor on the cell survival rate (preliminary experiments and regular controls within the experiments; data not shown). Control cells were only treated with medium. The proportion of treated cells in relation to untreated controls was determined 5, 24, 48 and 96 h after the beginning of the drug exposure. The medium for 96-h experiments was renewed before cell treatment (24 h after inoculation). The medium-compound mix was discarded at the time points mentioned above and cells were fixed with 2% glutaraldehyde (Sigma-Aldrich) in PBS for 20 min. Glutaraldehyde was removed and cells were dyed for 30 min with 0.1% crystal violet (Roth GmbH, Karlsruhe, Germany) in deionized water. After washing with deionized water, the plates were air-dried. Subsequently, crystal violet was solubilized out of the cells by adding 2% Triton X-100 (Sigma-Aldrich, Steinheim, Germany) in deionized water. After 1 h of incubation, absorbance was measured at 570 nm using a 96-well microtiter plate reader (MRX microplate reader, Dynatech Laboratories, El Paso, US). Experiments were performed in six to eight biological replicates with two technical replicates for each combination of cell type, incubation time and pharmacologic compound concentration. The ratios of mean optical density of the duplicate to mean optical density of the associated controls were used for dose-response curves.

#### Cytotoxicity assays

The cytotoxicity of BA was evaluated using the CellTiter 96® AQ_ueous_ One Solution Cell Proliferation Assay (MTS) (Promega GmbH, Mannheim, Germany). Cells were seeded into 96-well microtiter plates with the appropriate cell densities to achieve confluence after 48 h (MelDuWi 30.000 cells/well; PriFi1, PriFi2, eRGO1 20.000 cells/well). After 48 h, these cells were treated in accordance with the CVS assay. Experiments were stopped at the same time points as the CVS assay. The medium for the 96-h experiments was renewed before treatment. The MTS was applied in accordance with the manufacturer’s instructions. After 1 h incubation, the plate absorbance was measured at 490 nm using a 96-well microtiter plate reader (MRX microplate reader, Dynatech Laboratories, El Paso, US). Experiments were performed in six to eight biological replicates with two technical replicates for each combination of pharmacologic compound, cell type, incubation time and concentration.

### Diffusion of betulinic acid into equine skin

#### Skin samples

Skin samples from seven adult horses of different sex (three mares, two geldings, two unknown) and breed (including three Warmbloods, one Icelandic horse and one Welsh Cob pony, two unknown) were harvested at the Institute of Pathology, University of Veterinary Medicine Hannover, Foundation, Hannover, after euthanasia at the Clinic for Horses, University of Veterinary Medicine Hannover, Foundation, Hannover, for reasons unrelated to the present study. The horses’ ages ranged from 4 to 24 years, with a median of 13.5 years. Skin from the lateral thorax was dissected and stored at − 20 °C for up to 5 months.

#### Drug formulation

“Basiscreme DAC” (pharmaceutical amphiphilic formulation as published in the German Drug Codex) with 1% BA and 20% medium-chain triglycerides was provided by Skinomics GmbH, Halle, Germany.

#### In vitro permeation

In order to investigate the penetration and permeation of 1% BA with 20% medium-chain triglycerides in “Basiscreme DAC” through equine skin, the skin samples were defrosted overnight at room temperature. The coat was clipped to a length of approximately 0.5 mm. The integrity of the skin samples was visually assessed. Skin slices of 800 μm (+/− 110 μm) thickness were obtained with an electrical dermatome (Zimmer, Eschbach, Germany). Franz-type diffusion cells (FDC) (PermeGear, Riegelsville, USA, and Gauer Glas, Püttlingen, Germany) with a diffusion area of 1.77 cm^2^ and an acceptor chamber volume of approximately 12 mL were filled with PBS and 1% bovine serum albumin. The acceptor chamber content was constantly stirred with a magnetic stirrer at 500 rpm. Diffusion chambers were maintained at 34 °C to ensure a skin temperature of 32 ± 0.5 °C. Before use, equal hydration of the skin samples was obtained by 30 min immersion in PBS. After gently drying with a paper tissue, 20 mg of the drug formulation was carefully applied to the skin surface (*stratum corneum)* covering the complete diffusion area before mounting the skin pieces onto the FDC. The donor chamber and sampling tube were covered with parafilm.

#### Terminal procedures and BA quantification

After 24 h, the remaining donor formulation was removed from the skin with a dry cotton swab. Cotton swabs, acceptor medium and exposed areas of the skin samples, which were cut out with a scalpel blade, were stored at − 20 °C until further processing and analysis. In order to determine the amount of BA in different skin layers, frozen skin samples were fixed on tissue freezing medium (Leica Biosystems Nussloch GmbH, Nussloch, Germany) and placed in a cryostat (CryoStar™ NX70 Cryostat, Thermofisher, Darmstadt, Germany). From each skin sample slices were cut horizontally to the epidermis, starting with the *stratum corneum* side uppermost, and stored separately. While the first slice had a thickness of 10 μm the following slices were 20 μm thick. After reaching a skin depth of 310 μm, slices were pooled at 5 × 20 μm until a depth of a maximum of 910 μm was reached. The blade was cleaned with tissues soaked in 70% ethanol (CG Chemikalien, Laatzen, Germany) between each cut. These cleaning tissues and skin samples were stored at − 20 °C until final analysis. An analytic high-performance liquid chromatography method was developed for BA quantification in the different skin layers, acceptor medium and cleaning utensils mentioned previously. Reverse phase analysis was performed using an Agilent 1100 system (Agilent, Waldbronn, Germany) on a Kinetex column (5 μm, C18, 100 Å, 250 × 4.6 mm; Phenomenex, Torrance, US) at 35 °C developing with acetonitrile:water (0.1% HCOOH) 4:1 (v/v) at 2.5 mL/min. The diode array detector was set at 200 nm.

### Statistical analysis

Technical duplicates with a coefficient of variation of more than 20% were excluded from the analysis of the cell assays. The pharmacodynamic model 108 of Phoenix® WinNonlin® software (version 8.1, Certara, USA) was used to determine half-maximal inhibitory concentrations (IC_50_ values). Further statistical analysis was performed with R 3.5.1 [60]. A generalized additive model was fitted for both MTS and CVS using the ‘mgcv’ package [61] to estimate the effects of the BA concentration, cell line and duration of incubation on the ratio of the mean optical density of the duplicates from six to eight replicates to the mean optical density of the associated controls. The effect of concentration was modelled as a smoothed term interacting with the duration of incubation using a thin plate regression spline. The *P*-values were obtained by performing a Wald test for each parameter. Post-hoc comparisons for the cell line and duration of incubation were performed using the ‘multcomp’ package with single-step adjustment of the P-values [62]. Plots were produced with ggplot2 [63]. Statistical significance was set at *P* < 0.05.

## Data Availability

The datasets analyzed during the current study are available from the corresponding author on reasonable request.

## Abbreviations

BA: Betulinic acid
CVS: Crystal violet staining assay
DAC: Deutscher Arzneimittel Codex (German Drug Codex)
DMSO: Dimethyl sulfoxide
EMM: Equine malignant melanoma
FBS: Fetal bovine serum
FDC: Franz-type diffusion cell
IC_50_: Half-maximal inhibitory concentration
MTS: CellTiter 96® AQ_ueous_ One Solution Cell Proliferation Assay (Promega)
PBS: Phosphate-buffered saline
rpm: rounds per minute
